# Antibiotic Knowledge and Non-Prescription Antibiotic Use Among Jordanian Adults: A Community-Based Cross-Sectional Study

**DOI:** 10.3390/antibiotics15101017

**Published:** 2026-10-14

**Authors:** Yousef Al-Hajaya, Shadi Mustafa, Ma’en Al-Odat, Amane Alaroud, Enas Almanasreh, Muna M. Abbas, Yasmeen Hazaimeh, Mais Hijazi, Anwar Kandari

**Affiliations:** 1 Department of Biological Sciences, Faculty of Science, Mutah University, Mutah 61710, Jordan; 2 Faculty of Pharmacy, Jerash University, Jerash 26150, Jordan; shadi.mustafa@outlook.com; 3 Department of Medical Laboratory Science, Faculty of Allied Medical Science, Mutah University, Mutah 61710, Jordan; maen.alodat@mutah.edu.jo; 4 Faculty of Pharmacy, Mutah University, Mutah 61710, Jordanenas.almanasreh@mutah.edu.jo (E.A.); yasmeenahmad424@gmail.com (Y.H.); 5 Department of Allied Medical Sciences, Zarqa University College, Al-Balqa Applied University, Salt 19117, Jordan; muna.abbas@bau.edu.jo; 6 Faculty of Pharmacy, Jordan University of Science and Technology, Irbid 22110, Jordan; phmaishijazi@gmail.com; 7 Dasman Diabetes Institute, Kuwait City 15462, Kuwait; anwar.kandari@dasmaninstitute.org

**Keywords:** antimicrobial resistance, antibiotic misuse, antibiotic stewardship, self-medication, public awareness, community survey, Jordan

## Abstract

**Background**: Antimicrobial resistance (AMR) is one of the most significant current public health issues, which is driven crucially by the misuse and overuse of antibiotics. Antibiotics are still available without a prescription in many underdeveloped countries, including Jordan, allowing for self-medication and incorrect usage, which contributes to the establishment and spread of resistant bacteria. This cross-sectional study aimed to evaluate antibiotic-use practices and assess knowledge and awareness of antimicrobial resistance among the Jordanian population. **Methods**: We conducted a cross-sectional observational survey between September 2023 and January 2025, targeting males and females in Jordan. A QR code was used to distribute an online convenience sampling strategy with 57 questions across social media channels. The survey investigated a variety of aspects, including demographic traits, antibiotic knowledge and behaviour, and public awareness of resistance. The questionnaire was completed by 1031 people. The data was analysed using descriptive statistics. The chi-square test was performed to compare demographic groupings, and *p* < 0.05 was considered significant. **Results**: Among the 1031 participants, 61.3% were female and 37.8% were male. Recent utilization of antibiotics was prevalent, with 37.8% indicating usage in the preceding month and 26.4% within the last six months. It is noteworthy that 61% (*n* = 624) of participants indicated that they had consumed antibiotics without a prescription, while 33.6% (*n* = 345) reported acquiring antibiotics for children without a prescription. Furthermore, pharmacies constituted the predominant source of antibiotics, accounting for 90.4% of the total. Common misconceptions regarding the indications for antibiotics were observed, with a segment of participants under the impression that antibiotics are effective in treating symptoms such as fever (40.6%) and body pain (15%). Additionally, participants exhibited a moderate level of awareness regarding antimicrobial resistance, with 69.2% demonstrating familiarity with the concept, while 90% acknowledged the increased difficulty in treating infections caused by antimicrobial-resistant organisms. Nevertheless, major misconceptions persisted, including 84.8% incorrectly believing that antibiotic resistance occurs when the human body becomes resistant to antibiotics and then antibiotics cannot work properly. More than half of the respondents (*n* = 569; 57.1%) also believed that AMR only or mainly affects individuals who frequently take antibiotics and does not concern others. **Conclusions**: This study has identified that non-prescription antibiotic use remains prevalent in Jordan and is accompanied by substantial misconceptions regarding antimicrobial resistance. Although general awareness of the seriousness of AMR was relatively high, important knowledge gaps persisted. In the adjusted analysis, among the covariates, participants aged 51–60 years had higher odds of reporting non-prescription antibiotic use than those aged 18–30 years (aOR = 1.872, 95% CI 1.072–3.268; *p* = 0.028). Moreover, participants with a high-school education (aOR = 0.415, 95% CI 0.196–0.879; *p* = 0.022) and those with a master’s degree (aOR = 0.411, 95% CI 0.181–0.933; *p* = 0.034) had lower odds than the no-formal-education reference group. No other covariate was statistically significant. The findings clearly indicate the need for strengthened enforcement of prescription-only antibiotic dispensing regulations, targeted public education to address specific misconceptions, and expanded antimicrobial stewardship initiatives involving community pharmacists to promote responsible antibiotic use and lessen the growing threat of antimicrobial resistance.

## 1. Introduction

Antimicrobial resistance (AMR) is widely recognized as one of the greatest threats to global public health. The emergence and spread of resistant microorganisms reduce the effectiveness of antimicrobial therapies, resulting in treatment failure, prolonged illness, increased healthcare costs, and higher mortality rates [1,2,3,4]. In 2019, bacterial AMR was estimated to be directly responsible for 1.27 million deaths and associated with approximately 4.95 million deaths worldwide [5]. Without effective interventions, the burden of AMR is expected to rise considerably, with projections indicating that antimicrobial-resistant infections may contribute to nearly 39 million deaths worldwide between 2025 and 2050 [6,7]. The growing burden of AMR threatens many advances in modern medicine, including surgical procedures, organ transplantation, cancer chemotherapy and intensive care management [6].

Antibiotics have revolutionized the treatment of bacterial infections and significantly improved patient outcomes [8]. However, their inappropriate use has led to the emergence and spread of antibiotic-resistant bacteria [9]. The development of AMR has been linked to several practices including inappropriate antibiotic use, self-medication, purchasing antibiotics without a prescription, using antibiotics for viral infections, sharing medications and failing to complete prescribed treatment courses [9,10,11]; therefore, AMR has become a major contributor to increased morbidity, mortality, and healthcare costs worldwide.

The Middle East and North Africa (MENA) region encounters significant obstacles in managing antimicrobial resistance. AMR is on the rise throughout the MENA region, accompanied by an increasing prevalence of multidrug-resistant pathogens. These pathogens present a significant challenge to healthcare systems, efforts in antimicrobial stewardship, and overall public health outcomes [12,13]. The consumption of antibiotics, self-medication practices, the accessibility of antibiotics without prescriptions, insufficient adherence to pharmaceutical regulations, low public awareness, and challenges in accessing healthcare have exacerbated the misuse of antibiotics, leading to the rise in antimicrobial resistance (AMR) [14]. Recent studies from the MENA region have examined settled misconceptions and knowledge regarding antibiotic use, including inappropriate self-medication practices, misuse of antibiotics for non-bacterial illnesses, and poor adherence to recommended treatment guidelines [15,16,17,18,19]. These concerns have been particularly documented specifically for common respiratory diseases and conditions that do not require antimicrobial treatment [20,21,22,23,24].

In Jordan, despite regulations restricting the dispensing of antibiotics to prescription-only use, non-prescription antibiotic consumption remains common [25,26,27]. Previous studies have shown that antibiotics can be obtained directly from community pharmacies without medical consultation, fostering self-medication behaviours [28]. Several factors have been related to antibiotic self-medication in Jordan, including prolonged waiting times for physician consultations, the cost of healthcare services, previous experiences with antibiotic use, and the perception that common illnesses do not require professional medical advice [26,28,29]. Furthermore, increasing antimicrobial consumption has been reported in Jordan, raising concerns regarding its potential contribution to AMR [30,31].

Although previous studies have evaluated antibiotic use and public awareness of AMR in Jordan, contemporary data assessing both antibiotic-use practices and public understanding of AMR remain limited [25,26]. Therefore, updated evidence is needed to assess current antibiotic-use practices and evaluate the level of public knowledge and awareness of AMR. Accordingly, the present study aimed to evaluate community practices regarding antibiotic consumption, assess knowledge of antibiotic indications, and investigate awareness and understanding of AMR among individuals in Jordan.

## 2. Materials and Methods

### 2.1. Sampling

A questionnaire was distributed over seventeen months, and survey completion was carried out between 18 September 2023 and 5 January 2025. The majority of participants filled out the survey in 2023 (*n* = 962) and a few participants filled out the survey in 2024 (*n* = 57) and 2025 (*n* = 12). A total of 1031 participants from the Jordanian community were recruited in this survey and completed the 57-question survey online using Google Forms (Tables S1 and S2). The survey was designed and carefully translated into the Arabic language. Then the survey was designed in the Google Forms platform (both versions were also provided in the Supplements). The distribution of the survey was done by generating a QR code for the survey link using an online convenience sampling approach. This QR code was sent to several social media groups such as WhatsApp or Facebook. The survey was performed for different classes of community in Jordan.

### 2.2. Development and Description of Survey Tool

The authors formulated the survey to identify the situation of antimicrobial consumption without a prescription. In this survey, the perspective of the community in Jordan was measured regarding the practice of antimicrobial consumption. The survey was designed to perform an assessment of community perspectives regarding antimicrobials using the evaluation of three critical domains of information: (1) the use of antimicrobials by individuals and the frequency of consumption of antimicrobials. (2) The community knowledge of antimicrobials and the diseases that can be treated by antimicrobials. (3) The community knowledge of AMR and its impact on a normal individual’s life and the threat that it could pose. A questionnaire aimed to evaluate the community knowledge and practices of antimicrobial use in Jordan.

The antibiotic knowledge score regarding appropriate antibiotic indications was assessed using seven items asking participants whether selected conditions could be treated with antibiotics. Each response was coded as correct (1 point) or incorrect and “Don’t know” (0 points), and the item scores were summed to produce a total antibiotic knowledge score ranging from 0 to 7, with higher scores indicating greater knowledge of appropriate antibiotic indications. This score corresponds to the score distribution was used as the primary predictor in the logistic regression analysis. The primary outcome for the regression analysis was self-reported non-prescription antibiotic use, based on the survey question asking participants whether they had consumed antibiotics without a prescription.

### 2.3. Data Analysis

Descriptive statistics were used to summarize participant characteristics, antibiotic-use practices, antibiotic knowledge, and awareness of antimicrobial resistance. Categorical variables are presented as frequencies and percentages. Associations between categorical variables were evaluated using Pearson’s chi-square test.

To investigate whether antibiotic knowledge was associated with self-reported non-prescription antibiotic use, binary logistic regression analyses were performed. The dependent variable was self-reported consumption of antibiotics without a prescription. The primary independent variable was the seven-item antibiotic knowledge score, which was treated as a continuous predictor. Crude odds ratios (ORs) were first estimated using univariable logistic regression. A multivariable logistic regression model was subsequently fitted, adjusting for sex, age group, region, monthly income, educational level, and marital status.

Missing observations were not imputed. The multivariable regression was conducted using complete-case analysis, including participants with complete data for the outcome, antibiotic knowledge score, and all included covariates.

Model performance and assumptions were assessed using the Hosmer–Lemeshow goodness-of-fit test, receiver operating characteristic area under the curve (ROC AUC), McFadden pseudo-R^2^, overall likelihood-ratio test, variance inflation factors for multicollinearity, and assessment of linearity in the logit for the continuous antibiotic knowledge score. A sensitivity analysis was performed by recalculating the knowledge score after excluding the potentially ambiguous sore throat/tonsillitis item and repeating the adjusted regression analysis. Statistical significance was defined as *p* < 0.05.

Analyses were performed using IBM SPSS Statistics version 25.

## 3. Results

### 3.1. Study Participants

A total of 1031 participants completed the survey (however, item-level denominators vary due to missing data). Female participants accounted for almost 62% of the participants while males represented 38%. Most participants were in the 18–30 years age group, followed by 41–50 and 31–40 years. Most participants were from urban areas and settled in the northern governorates. Participants’ education was generally high (about half with a bachelor’s degree), and most participants were married (Table 1).

### 3.2. Community Antibiotics Consumption

Recent antibiotic exposure was common among participants; around two in five reported use in the past month, and a further group reported use within the past six months. A clear majority indicated receiving counselling from a pharmacist or physician on how to use antibiotics; despite this, non-prescription access was common, with a large portion reporting taking antibiotics without a prescription, and about one-third had bought antibiotics for a child without a prescription. Additionally, pharmacies were the predominant source of antibiotics whereas online purchases were rare (Table 2). Clearly, antibiotic use trends differed markedly according to participant age; this variation might be because of differences in knowledge and awareness of optimal antibiotic use. Furthermore, the variation in participants’ income manifested in variation in their response to antibiotic consumption. However, educational level was not significantly related to antibiotic intake.

### 3.3. Knowledge of Antibiotics

Substantial misconceptions among participants on the appropriate indications for antibiotic use were observed. While respondents commonly endorsed antibiotics for conditions likely to be bacterial (e.g., urinary tract infection), many also believed antibiotics treat conditions that are often viral or non-bacterial (e.g., measles, headache, body pain, and fever) (Table 3). Overall the participants had modest knowledge about the indications of antibiotics, with a minority scoring near perfect and a non-negligible minority scoring very low (Table 4). Statistical significance appeared between groups of the age of participants in their knowledge of the treatment of diseases with antibiotics. However, there is less significance between groups of participants by region, income and education.

To examine whether antibiotic knowledge was associated with non-prescription antibiotic use, a multivariable binary logistic regression analysis was performed with non-prescription antibiotic use as the dependent variable. After adjustment, the antibiotic knowledge score was not significantly associated with self-reported non-prescription antibiotic use (aOR = 1.006, 95% CI 0.936–1.080; *p* = 0.881). Among the covariates, participants aged 51–60 years had higher odds of reporting non-prescription antibiotic use than those aged 18–30 years (aOR = 1.872, 95% CI 1.072–3.268; *p* = 0.028). Moreover, participants with a high-school education (aOR = 0.415, 95% CI 0.196–0.879; *p* = 0.022) and those with a master’s degree (aOR = 0.411, 95% CI 0.181–0.933; *p* = 0.034) had lower odds than the no-formal-education reference group. No other covariate was statistically significant. (Tables S1 and S2).

### 3.4. Knowledge of Antimicrobial Resistance

Awareness of antimicrobial resistance was relatively high, with 69.2% of participants reporting awareness of the term. However, awareness of the term “superbug” was considerably lower, reported by only 29.7% of participants, and most recognized key consequences of AMR (harder-to-treat infections, rising prevalence, and increased risk around medical procedures). However, several misconceptions persisted—especially the idea that the human body (rather than bacteria) becomes resistant, that AMR is mainly a problem elsewhere, and that it predominantly concerns frequent antibiotic users. Despite these misconceptions, a majority perceived personal or family susceptibility (Table 5). Statistical significance was found between groups of participants in their region, income and education for their knowledge of AMR and resistant bacteria especially superbug bacteria. In contrast, gender and age of participants manifested less variation in their knowledge of AMR.

Participants demonstrated relatively high awareness of the clinical consequences of AMR, but important conceptual misconceptions remained. Demonstrated that more than half rejected the idea that resistance is only a problem in other countries; however, 41.6% still believed it is not a problem for Jordan. A larger gap appears in personal risk perception. A portion of 76.5% agreed that resistance can affect them and their families; 57.1% believed it concerns only people who frequently take antibiotics. The knowledge concerning the mode of pathogen transmission was split, with 51.6% being aware of the fact that resistant bacteria spread from one individual to another. The majority of individuals had an idea of the effects of these infections on the patient’s health. As many as 86.6% were aware that resistant infections make surgeries and transplants more threatening. Similarly, 86.3% recognized that resistance is increasing, and 89% identified misuse of antibiotics as a main cause. However, 84.8% incorrectly thought resistance occurs when the human body becomes resistant rather than the bacteria. These findings suggest that AMR awareness may be relatively high at the level of perceived severity, but remains incomplete at the level of mechanism, transmission, and personal risk (Table 6).

Very few participants scored at the lowest levels. Less than 2% scored one or two correct answers. Performance improved steadily across higher score categories. About 17.7% achieved seven correct answers, which represents the largest group. Another 12.9% scored eight correct answers, and 3.5% achieved nine correct answers. More than half of the participants scored six or more correct answers, which indicates a generally acceptable level of knowledge. However, around 31.1% scored five or fewer correct answers, which reflects a substantial knowledge gap in a notable segment of the sample (Table 7). The above findings indicate an average to decent level of awareness, although it is still necessary to introduce educational strategies to correct existing gaps.

## 4. Discussion

The current study presents new insights into the issue of antibiotic-use practices as well as antimicrobial resistance awareness among the Jordanian population. Despite the fairly high level of awareness of the issue of AMR, antibiotic misuse practices were prevalent. Over 50 percent of individuals indicated that they got antibiotics without a prescription. Moreover, many myths and misconceptions related to the use of antibiotics and AMR were widely spread.

Non-prescription antibiotic consumption continues to pose a crucial problem in Jordan. In the current study, 61% of the respondents admitted to taking antibiotics without prescriptions, and pharmacies constituted the main source for getting antibiotics. These results agree with the results obtained in previous studies done in Jordan, which indicate high rates of self-medication practices with ready access to antibiotics at pharmacies [26,29,32]. The fact that many people continue to abuse antibiotic use without prescriptions indicates that current regulations are not enough and may not have a positive effect without proper monitoring of antibiotic use. Access, healthcare costs, convenience, and past experience with antibiotics may all contribute to such self-medication practices.

A number of misconceptions about antibiotic use were noted. More than 40% of participants believed that antibiotics could be used to treat fever, while a proportion of respondents believed that antibiotics could be used for conditions such as measles, headache and body pain. Such misconceptions may contribute to inappropriate antibiotic use, particularly for viral and self-limiting illnesses, thereby increasing the risk of unnecessary antibiotic exposure and antimicrobial resistance [18]. Similar misconceptions have been reported in previous studies from Jordan and neighbouring countries, indicating that misunderstanding of appropriate antibiotic indications remains a persistent public health challenge in the region [18,33,34].

After adjustment, the antibiotic knowledge score was not significantly associated with self-reported non-prescription antibiotic use (aOR = 1.006, 95% CI 0.936–1.080; *p* = 0.881). Among the covariates, participants aged 51–60 years had higher odds of reporting non-prescription antibiotic use than those aged 18–30 years (aOR = 1.872, 95% CI 1.072–3.268; *p* = 0.028). Moreover, participants with a high-school education (aOR = 0.415, 95% CI 0.196–0.879; *p* = 0.022) and those with a master’s degree (aOR = 0.411, 95% CI 0.181–0.933; *p* = 0.034) had lower odds than the no-formal-education reference group. No other covariate was statistically significant. The high proportion of participants who believed that antibiotics can be used for sore throat or tonsillitis should be interpreted cautiously. In the present study, the antibiotic knowledge score was not significantly associated with self-reported non-prescription antibiotic use after adjustment for demographic characteristics. The confidence interval was compatible with only relatively small associations in either direction. Therefore, these findings should not be interpreted as demonstrating that knowledge has no influence on antibiotic-use behaviour. Rather, the study did not identify statistically significant evidence of an association between the measured knowledge score and non-prescription antibiotic use in this sample. Bacterial tonsillitis may require antibiotic therapy, whereas most sore throat cases are viral and do not require antibiotics [35,36]. Importantly, exclusion of the sore throat/tonsillitis item from the antibiotic knowledge score did not materially change the regression result. In the sensitivity analysis using the six-item score, antibiotic knowledge remained non-significantly associated with non-prescription antibiotic use (adjusted OR = 1.020, 95% CI: 0.948–1.098; *p* = 0.588). This finding indicates that the primary regression conclusion was not dependent on the classification of this potentially ambiguous item (Table S3).

Descriptively, relatively high awareness of AMR coexisted with frequent reports of non-prescription antibiotic use. However, these aggregate descriptive findings should not be interpreted as evidence of an individual-level awareness–behaviour relationship because AMR awareness was not the primary predictor evaluated in the regression model. While 69.2% of participants reported awareness of AMR and most recognized that resistant infections are more difficult to treat, inappropriate antibiotic-use behaviours remained common. The coexistence of AMR awareness and non-prescription antibiotic use at the sample level warrants further investigation using study designs specifically developed to examine individual-level relationships between knowledge, awareness, and antibiotic-use behaviour [37]. Factors such as easy antibiotic availability, convenience, healthcare costs, previous experiences with antibiotics, and misconceptions regarding their appropriate use may continue to drive inappropriate antibiotic consumption despite awareness of AMR. These findings support previous observations that improving awareness alone may be insufficient to reduce antibiotic misuse without addressing behavioural, social and structural factors that influence antibiotic access and consumption [35].

Despite the relatively high level of AMR awareness observed in this study, important conceptual misunderstandings persisted. Notably, 84.8% of participants incorrectly believed that antibiotic resistance occurs when the human body becomes resistant to antibiotics rather than the bacteria themselves. Similar misconceptions have been reported previously and may hinder public understanding of how inappropriate antibiotic use contributes to the development and spread of resistant bacteria [38]. In addition, more than half of the respondents thought that antimicrobial resistance mostly affects people who take antibiotics regularly, implying that many of them fail to realize the full extent of its public health ramifications. This means that there is a high probability of many people thinking of AMR as a problem at an individual level, thereby overlooking the fact that resistant microorganisms can easily spread from one person to another. This could be an impediment to efforts to prevent antimicrobial resistance.

The observed misconceptions regarding antibiotic use and AMR highlight potential areas for targeted public health education. Such educational approaches may be considered alongside regulatory and antimicrobial stewardship strategies aimed at promoting appropriate antibiotic use [39,40]. Pharmacists are well-positioned to discourage inappropriate antibiotic requests, provide patients with information on the proper use of antibiotics, refer patients to seek medical attention, and disseminate other antimicrobial stewardship messages [41,42].

## 5. Conclusions

The current cross-sectional survey offers recent findings on antibiotic use practices and awareness of AMR in the Jordanian community. Although there seems to be an acceptable level of awareness regarding the significance of AMR, inappropriate antibiotic usage is still prevalent, characterized by self-medication practices and incorrect perceptions about the indication for antibiotics as well as the mechanism of action. Although non-prescription antibiotic use and misconceptions regarding antibiotic indications were common, the adjusted analysis found no statistically significant evidence of an association between the antibiotic knowledge score and self-reported non-prescription antibiotic use. This finding remained consistent after excluding the potentially ambiguous sore throat/tonsillitis item from the knowledge score. Given the cross-sectional design, these findings should not be interpreted as demonstrating either the presence or absence of a causal relationship between antibiotic knowledge and antibiotic-use behaviour.

Given the availability of antibiotics over-the-counter and the misconceptions around their use, it becomes imperative for the strengthening of pharmaceutical laws and the implementation of antimicrobial stewardship programmes. While public health efforts will have to emphasize raising awareness about AMR, emphasis should also be placed on correcting misconceptions and promoting proper antibiotic use. The active participation of community pharmacists in antimicrobial stewardship is essential through patient education and proper referrals.

Overall, the findings emphasize the need for a comprehensive approach that integrates public education, regulatory enforcement, and pharmacist-led stewardship efforts to reduce inappropriate antibiotic use and mitigate the growing threat of AMR in Jordan. Future studies should investigate individual-level relationships between antibiotic knowledge, AMR awareness, and antibiotic-use practices in Jordan and other countries in the MENA region using designs that allow these relationships to be evaluated directly.

## 6. Limitation

The data obtained from this study need to be understood in light of certain limitations. The general term “antibiotic” may vary between participants and may differ in their understanding of which medications are classified as antibiotics, potentially resulting in misclassification and contributing to some incorrect responses. Moreover, future surveys should distinguish between viral sore throat and confirmed bacterial tonsillitis to assess this misconception more accurately. Also, since the design was cross-sectional in nature, no relationship can be derived between the level of awareness among people and their behaviours when using antibiotics. Moreover, conducting the study using an online convenience sampling strategy may have caused some form of selection bias and may not accurately reflect the views held by all the different demographics within the Jordanian population. In addition, the majority of the subjects who participated in this research study came from the northern part of Jordan (79.2%). Another limitation to this study is that responses were self-reported and may, therefore, be subject to recall bias or social desirability bias. However, this study offers up-to-date insights on antibiotic practices and the level of awareness regarding AMR in Jordan. In addition, future studies should specifically examine individual-level relationships between different dimensions of antibiotic knowledge, AMR awareness, and antibiotic-use behaviours using validated measures and longitudinal or other appropriate study designs. Although data collection extended from September 2023 to January 2025, most responses (93.3%) were obtained during 2023. The relatively small number of participants recruited in 2024 and 2025 limited our ability to assess potential temporal differences. Therefore, some degree of temporal heterogeneity cannot be excluded. The multivariable analysis used complete-case analysis, resulting in 878 of the 1031 participants being included in the primary adjusted model. Although this approach avoids assumptions required for imputing missing observations, exclusion of participants with incomplete data may introduce bias if missingness is systematically related to the study variables.

## Figures and Tables

**Table 1:** Descriptive analysis for the participants in this study.

Categories	Total (%)
Gender	
Male	390 (37.8%)
Female	632 (61.3%)
Missing	9 (0.9%)
Age	
18–30	336 (32.59%)
31–40	247 (23.96%)
41–50	269 (26.09%)
51–60	143 (13.87%)
>60	35 (3.39%)
Missing	1 (0.10%)
Region	
North Region	814 (79.0%)
Middle region	157 (15.2%)
South Region	57 (5.5%)
Missing	3 (0.3%)
Income	
<300 JOD monthly	313 (30.4%)
300–600 JOD monthly	386 (37.4%)
601–1000 JOD monthly	205 (19.9%)
1000–2000 JOD monthly	75 (7.3%)
>2000 JOD monthly	26 (2.5%)
Missing	26 (2.5%)
Education	
No education	53 (5.1%)
High school	143 (13.9%)
Diploma	165 (16%)
Bachelor	531 (51.5%)
Master	92 (8.9%)
Doctorate	45 (4.4%)
Missing	2 (0.2%)
Environment	
Rural	237 (23.0%)
Urban	789 (76.5%)
Missing	5 (0.5%)
Marital status	
Single	315 (30.6%)
Married	668 (64.8%)
Divorce	25 (2.4%)
Widow	21 (2.0%)
Missing	2 (0.2%)

**Table 2:** Participants’ antibiotic use practices and associations with sociodemographic characteristics.

Questions	Valid *n*	Missing *n*	*n* (%)	*p*-Value					
				Gender	Age	Region	Income	Education	Marital Status
Last time antibiotics were taken	1026	5		0.395	0.581	0.398	0.089	0.007 **	0.668
Last month			388 (37.8)						
Within the last 6 months			271 (26.4)						
Within the last year			136 (13.3)						
More than 1 year ago			193 (18.8)						
Never taken an antibiotic			38 (3.7)						
Received counselling on antibiotic use	1028	3		0.166	0.039 *	0.758	<0.001 **	0.108	<0.001 **
Yes			765 (74.4)						
No			263 (25.6)						
Consumed antibiotics without prescription	1025	6		0.888	0.038 *	0.144	0.659	0.136	0.517
Yes			624 (60.9)						
No			401 (39.1)						
Purchased antibiotics for a child without a prescription	1026	5		0.536	<0.001 **	0.01 **	<0.001 **	0.058	<0.001 **
Yes			345 (33.6)						
No			681 (66.4)						
Source of antibiotics	1021	10		0.35	0.055	0.675	0.012 *	0.08	0.208
Pharmacy/medical store			923 (90.4)						
Internet			2 (0.2)						
Friend or relative			31 (3.0)						
Previously prescribed/retained antibiotic			65 (6.4)						

Note: Percentages were calculated using valid responses for each survey item as the denominator. Missing responses were excluded from percentage calculations and are reported separately. Associations were evaluated using Pearson’s chi-square test. *p* < 0.05 was considered statistically significant. The significant values were underscored and bolded. Several antibiotic-use practices differed significantly across demographic groups in the bivariate analyses. Non-prescription antibiotic use differed by age group (*p* = 0.038), while purchasing antibiotics for children without a prescription differed by age, region, income, and marital status (all *p* < 0.05). Receipt of counselling also varied according to age, income, and marital status. * indicate significance *p* < 0.05, ** indicate highly significance *p* < 0.001.

**Table 3:** Participants’ knowledge regarding conditions that may be treated with antibiotics and associations with sociodemographic characteristics.

Condition			Yes (%)	No/Don’t Know (%)	*p*-Value					
					Gender	Age	Region	Income	Education	Marital Status
Fever	Valid data	*n* = 982	399 (40.6)	583 (59.4)	0.019 *	<0.001 **	0.011 *	<0.001 **	0.687	<0.001 **
	Missing data	49								
Sore throat/tonsillitis	Valid data	*n* = 1015	915 (90.1)	100 (9.9)	<0.001 **	0.048 *	0.294	0.007 **	0.881	<0.001 **
	Missing data	16								
Measles	Valid data	*n* = 963	137 (14.2)	826 (85.8)	0.144	0.039 *	0.402	<0.001 **	0.002 **	0.829
	Missing data	68								
Body pain	Valid data	*n* = 965	145 (15.0)	820 (85.0)	0.153	<0.001 **	0.105	<0.001 **	<0.001 **	<0.001 **
	Missing data	66								
Headache	Valid data	*n* = 960	117 (12.2)	843 (87.8)	0.005 **	<0.001 **	0.920	<0.001 **	0.003 **	<0.001 **
	Missing data	71								
Acquired immunodeficiency syndrome (AIDS)	Valid data	*n* = 965	109 (11.3)	856 (88.7)	0.171	<0.001 **	0.057	0.144	<0.001 **	<0.001 **
	Missing data	66								
Urinary tract infection	Valid data	*n* = 1004	788 (78.5)	216 (21.5)	0.012 *	<0.001 **	0.500	<0.001 **	0.522	<0.001 **
	Missing data	27								

Note: Percentages were calculated from valid responses for each item. “No/Do not know” combines participants who selected either “No” or “Do not know.” Missing responses were excluded from percentage calculations. The sore throat/tonsillitis item is clinically ambiguous because the questionnaire did not distinguish viral sore throat from confirmed bacterial pharyngitis/tonsillitis; it was excluded in the sensitivity analysis. Associations were evaluated using Pearson’s chi-square test. *p* < 0.05 was considered statistically significant. The significant values were underscored and bold. * indicate significance *p* < 0.05, ** indicate highly significance *p* < 0.001.

**Table 4:** Distribution of the antibiotic knowledge score based on participants’ knowledge of appropriate antibiotic indications.

Antibiotic Knowledge Score	*n* (%)
Missing data in any of the 7 items	104 (10.1)
0	42 (4.1)
1	90 (8.7)
2	76 (7.4)
3	117 (11.3)
4	199 (19.3)
5	194 (18.8)
6	195 (18.9)
7	14 (1.4)
Total	1031 (100.0)

Note: The score was calculated from seven antibiotic-indication items. Each correct response was assigned 1 point and each incorrect and “Don’t know” response was assigned 0 points (range 0–7). Higher scores indicate greater knowledge. This score was used as the primary predictor in the logistic regression. A sensitivity analysis excluded sore throat/tonsillitis.

**Table 5:** Participants’ awareness of antimicrobial resistance and associations with sociodemographic characteristics.

Awareness item			Yes (%)	No/Don’t Know (%)	*p*-Value					
					Gender	Age	Region	Income	Education	Marital Status
Awareness of antimicrobial resistance	Valid data	*n* = 1019	705 (69.2)	314 (30.8)	0.944	0.091	0.025 *	0.053	<0.001 **	0.011 *
	Missing data	12								
Awareness of antibiotic-resistant bacteria	Valid data	*n* = 1008	656 (65.1)	352 (34.9)	0.518	0.640	0.042 *	0.058	<0.001 **	<0.001 **
	Missing data	23								
Awareness of “superbug”	Valid data	*n* = 1003	298 (29.7)	705 (70.3)	0.628	0.002 **	0.001 **	0.071	<0.001 **	<0.001 **
	Missing data	28								

Note: Percentages were calculated using valid responses for each item. “No/Do not know” combines participants who selected either “No” or “Do not know.” Missing responses were excluded from percentage calculations. Associations were evaluated using Pearson’s chi-square test. *p* < 0.05 was considered statistically significant. The significant values were underscored and bold. * indicate significance *p* < 0.05, ** indicate highly significance *p* < 0.001.

**Table 6:** Participants’ responses to statements regarding antimicrobial resistance.

Statement			Correct *n* (%)	Incorrect *n* (%)
Antibiotic resistance is a problem in other countries, but not a problem for Jordan.	Valid data	*n* = 998	583 (58.4)	415 (41.6)
	Missing data	*n* = 33		
Antibiotic resistance is only a problem for people who frequently take antibiotics and does not concern others.	Valid data	*n* = 996	427 (42.9)	569 (57.1)
	Missing data	*n* = 35		
Antibiotic-resistant bacteria can be transmitted from person to person.	Valid data	*n* = 993	512 (51.6)	481 (48.4)
	Missing data	*n* = 38		
The problem of antibiotic resistance can affect me and my family.	Valid data	*n* = 995	761 (76.5)	234 (23.5)
	Missing data	*n* = 36		
Infections caused by antibiotic-resistant bacteria make medical procedures such as surgery, organ transplantation, and cancer treatment more dangerous and life-threatening.	Valid data	*n* = 987	855 (86.6)	132 (13.4)
	Missing data	*n* = 44		
Treating infections caused by antibiotic-resistant bacteria is more difficult.	Valid data	*n* = 982	884 (90.0)	98 (10.0)
	Missing data	*n* = 49		
Antibiotic-resistant bacteria are continually increasing.	Valid data	*n* = 987	852 (86.3)	135 (13.7)
	Missing data	*n* = 44		
Antibiotic resistance occurs when the human body becomes resistant to antibiotics and the antibiotic can no longer work properly.	Valid data	*n* = 983	149 (15.2)	834 (84.8)
	Missing data	*n* = 48		
Irresponsible use of antibiotics is a main cause of the emergence of antibiotic-resistant bacteria.	Valid data	*n* = 986	878 (89)	108 (11)
	Missing data	*n* = 45		

Note: Percentages were calculated using valid responses to each statement as the denominator. Missing responses were excluded from percentage calculations and are reported separately.

**Table 7:** Distribution of the antimicrobial resistance knowledge score among study participants (*n* = 1031).

AMR Knowledge Score	*n* (%)
Missing data in any of the 9 items	120 (11.6)
1	2 (0.2)
2	18 (1.7)
3	42 (4.1)
4	97 (9.4)
5	162 (15.7)
6	239 (23.2)
7	182 (17.7)
8	133 (12.9)
9	36 (3.5)
Total	1031 (100.0)

Note: The AMR knowledge score summarizes responses to the nine antimicrobial-resistance knowledge statements. This score is distinct from the seven-item antibiotic knowledge score in Table 4 and was not used as the primary predictor in the main logistic regression analysis.

## Data Availability

The datasets generated and/or analyzed during the current study are available from the corresponding author on reasonable request.

## Supplementary Materials

The following supporting information can be downloaded at: https://www.mdpi.com/article/10.3390/antibiotics15101017/s1, File S1: English translated version of questionnaire. File S2: Arabic version of questionnaire. Table S1: Regression table of the antibiotic knowledge score based on participants’ knowledge of appropriate antibiotic indications. Table S2: The model diagnostics table. Table S3: Model diagnostics/sensitivity table for sore throat evaluation.
