# Supplementary material for: Antibiotic Knowledge and Non-Prescription Antibiotic Use Among Jordanian Adults: A Community-Based Cross-Sectional Study
Source: Antibiotics (Basel). 2026 Oct 14;15(10):1017. doi: 10.3390/antibiotics15101017 (PMC13700557; doi:10.3390/antibiotics15101017)
Supplement: Supplementary file 1 [file antibiotics-15-01017-s001.zip › files1-community awarness AM survey English.pdf]

Q01. What is your gender?

Male

Female

Q02. What is your current age?

18–30

31–40

41–50

51–60

60 or above

Q03. Which governorate are you from?

Irbid

Ajloun

Jerash

Mafraq

Amman

Balqa

Zarqa

Madaba

Azraq

Tafila

Ma’an

Aqaba

Karak

Q04. Approximately, which of the following describes your monthly income?

Less than 300 JOD per month

300–600 JOD per month

601–1000 JOD per month

1000–2000 JOD per month

More than 2000 JOD per month

Q05. What is the highest level of education you have attained?

No formal education; did not pass General Secondary (Tawjihi)

General Secondary Education (Tawjihi)

Diploma

Bachelor’s

Master’s

Doctorate (PhD)

Q06. Do you work in the health field or as a healthcare provider (e.g., physician, lab technician, pharmacist, nurse, etc.)?

Yes

No

Q07. Which of the following best describes your place of residence?

Rural/Badia

City

Q08. Marital status:

Single

Married

Divorced

Widowed

Q09. Do you have children under 16 years of age?

Yes

No

Q10. When was the last time you took an antibiotic?

Within the last month

Within the last 6 months

Within the last year

More than a year ago

I have not taken an antibiotic

Q11. Did you receive advice from a pharmacist or physician on how to take the antibiotic?

Yes

No

Q12. Have you taken an antibiotic without a prescription?

Yes

No

Q13. Have you purchased an antibiotic for a child without a prescription?

Yes

No

Q14. Where did you obtain the antibiotic?

Pharmacy or medical supply store

Internet

Friend or family member

I kept leftover antibiotics from a previous illness

Q15. I often take antibiotics without a prescription.

Strongly agree

Agree

Neutral

Disagree

Strongly disagree

Q16. When do you think one should stop taking an antibiotic?

When you feel better

After completing the full course as prescribed by the doctor

I don’t know

Q17. Do you think the following condition can be treated with antibiotics? — Fever and high body temperature

Yes

No

Don't know

Q18. Do you think the following condition can be treated with antibiotics? — Sore throat and tonsillitis

Yes

No

Don't know

Q19. Do you think the following condition can be treated with antibiotics? — Measles

Yes

No

Don't know

Q20. Do you think the following condition can be treated with antibiotics? — Body pain

Yes

No

Don't know

Q21. Do you think the following condition can be treated with antibiotics? — Headache

Yes

No

Don't know

Q22. Do you think the following condition can be treated with antibiotics? — Acquired Immunodeficiency Syndrome (AIDS)

Yes

No

Don't know

Q23. Do you think the following condition can be treated with antibiotics? — Urinary tract infection

Yes

No

Don't know

Q24. Have you heard the term: Antibiotic resistance?

Yes

No

Q25. Have you heard the term: Antibiotic-resistant bacteria?

Yes

No

Q26. Have you heard the term: “Superbug” bacteria?

Yes

No

Q27. Where did you hear the term “antibiotic resistance”?

Healthcare provider (physician, pharmacist, nurse, etc.)

Media (newspapers, TV, radio, social media)

Friend or family member

I have not heard of antibiotic resistance before

Q28. Antibiotic resistance is a problem in other countries, not a problem for Jordan.

True

False

Q29. Antibiotic resistance is only a problem for people who frequently take antibiotics; it does not concern others.

True

False

Q30. Antibiotic-resistant bacteria can be transmitted from person to person.

True

False

Q31. The problem of antibiotic resistance can affect me and my family.

True

False

Q32. Infections caused by antibiotic-resistant bacteria make medical procedures (such as surgeries, organ transplants, and cancer treatment) more dangerous and life-threatening.

True

False

Q33. Treating antibiotic-resistant bacteria is more difficult.

True

False

Q34. Antibiotic-resistant bacteria are continually increasing.

True

False

Q35. Antibiotic resistance occurs when the human body becomes resistant to antibiotics and the antibiotic cannot work well.

True

False

Q36. Irresponsible use of antibiotics is a main cause of the emergence of antibiotic-resistant bacteria.

True

False

Q37. It is normal to take the same antibiotic that a friend or relative took if I have similar symptoms.

Strongly disagree

Disagree

Neutral

Agree

Strongly agree

Q38. Doctors should prescribe antibiotics only when needed.

Strongly disagree

Disagree

Neutral

Agree

Strongly agree

Q39. The government should provide financial incentives to pharmaceutical companies that produce a new antibiotic.

Strongly disagree

Disagree

Neutral

Agree

Strongly agree

Q40. Farmers should give fewer antibiotics to farm animals.

Strongly disagree

Disagree

Neutral

Agree

Strongly agree

Q41. People should not keep antibiotics for use in a later illness.

Strongly disagree

Disagree

Neutral

Agree

Strongly agree

Q42. It is normal to buy or ask a doctor for the same antibiotic if it helped me recover previously.

Strongly disagree

Disagree

Neutral

Agree

Strongly agree

Q43. People should take antibiotics only when prescribed by a doctor.

Strongly disagree

Disagree

Neutral

Agree

Strongly agree

Q44. Pharmaceutical companies should develop new antibiotics.

Strongly disagree

Disagree

Neutral

Agree

Strongly agree

Q45. Antibiotic resistance is one of the biggest problems facing the world.

Strongly disagree

Disagree

Neutral

Agree

Strongly agree

Q46. Medical experts will solve the problem of antibiotic resistance before it becomes a serious issue.

Strongly disagree

Disagree

Neutral

Agree

Strongly agree

Q47. Everyone should take responsibility for using antibiotics only when needed.

Strongly disagree

Disagree

Neutral

Agree

Strongly agree

Q48. I cannot make a difference in stopping antibiotic resistance.

Strongly disagree

Disagree

Neutral

Agree

Strongly agree

Q49. I am worried about the impact of antibiotic resistance on my health and my family's health.

Strongly disagree

Disagree

Neutral

Agree

Strongly agree

Q50. There is no risk from antibiotic resistance if I take antibiotics correctly and as prescribed.

Strongly disagree

Disagree

Neutral

Agree

Strongly agree

Q51. Do you think there should be community awareness campaigns to educate the public about the proper use of antibiotics?

Yes

No

Q52. Are you willing to participate in and attend awareness campaigns about the dangers of antibiotic-resistant bacteria?

Yes

No

Q53. Do you know that handwashing and vaccination are ways to prevent the spread of antibiotic-resistant bacteria?

Yes

No

Q54. Are you aware of the term "One Health" (the integration of human, animal, and environmental health in public health), and that these sectors together reduce the risk posed by antibiotic-resistant bacteria?

Yes

No

Q55. Have you changed your pattern of antibiotic use based on your awareness of the dangers of antibiotic-resistant bacteria?

Yes

No

Q56. What is your reason for taking antibiotics without a prescription?

To treat an illness

To prevent illness

To reduce medical treatment costs

Q57. Usually, how do you get information about antibiotics and how to use them?

Specialized health authorities

Media (TV, radio, internet, etc.)

Family and friends
