# Supplementary material for: Antibiotic Knowledge and Non-Prescription Antibiotic Use Among Jordanian Adults: A Community-Based Cross-Sectional Study
Source: Antibiotics (Basel). 2026 Oct 14;15(10):1017. doi: 10.3390/antibiotics15101017 (PMC13700557; doi:10.3390/antibiotics15101017)
Supplement: Supplementary file 1 [file antibiotics-15-01017-s001.zip › files2-community awarness AM survey.pdf]

					ما هو جنسك؟	Q01
					ذكر	
					أنثى	
					ما هو عمرك الان؟	Q02
					18-30	
					31-40	
					41-50	
					51-60	
					60 فأكثر	
					من أي محافظة أنت؟	Q03
					اربد	
					عجلون	
					جرش	
					المفرق	
					عمان	
					البلقاء	
					الزرقاء	
					مادبا	
					الأزرق	
					الطفيلة	
					معان	
					العقبة	
					الكرك	
					تقريبا، اي من التالية تعبر عن دخلك الشهري؟	Q04
					اقل من 300 دينار اردني شهريا	
					دينار اردني شهريا 300-600	
					دينار اردني شهريا 601-1000	
					دينار اردني شهريا 1000-2000	
					أكثر من 2000 دينار اردني شهريا	
					ما هو اعلى تحصيل علمي حصلت عليه؟	Q05
					بدون تعليم، غير مجتاز للثانوية العامة (التوجيهي)	
					الثانوية العامة (التوجيهي)	
					دبلوم	
					بكالوريوس	
					ماجستير	
					الدكتوراه	
					هل تعمل في المجال الصحي، او مزود خدمه صحيه (مثال: طبيب، فني مختبر، صيدلاني او ممرض ... الخ)	Q06
					نعم	
					لا	
					أي من التاليه تصف مكان اقامتك؟	Q07
					أرياف\بادية	
					مدينة	
					الحالة الاجتماعية؟	Q08
					أعزب \ عزباء	

					متزوج \ متزوجة	
					مطلق \ مطلقة	
					ارمل \ ارملة	
					هل لديك اطفال تحت 16 عاما؟	Q09
					نعم	
					لا	
					متى اخر مرة تناولت مضاد حيوي؟	Q10
					منذ شهر	
					خلال فترة 6 شهور	
					خلال فترة سنة	
					أكثر من سنه	
					لم اتناول مضاد حيوي	
			لا	نعم		
					هل حصلت على نصيحة حول كيفية تناول المضاد الحيوي من قبل صيدلاني او طبيب؟	Q11
					هل تناولت مضاد حيوي بدون وصفة طبية؟	Q12
					هل اشتريت المضاد الحيوي لطفل بدون وصفة طبية؟	Q13
					من اين حصلت على المضاد الحيوي؟	Q14
					صيدلية او متجر طبي	
					الانترنت	
					صديق او قريب عائلي	
					احتفظ بالمضاد الحيوي تناولته سابقا	
					في اغلب الاحيان اتناول المضاد الحيوي بدون وصفة طبية	Q15
					موافق بشدة	
					موافق	
					محايد	
					غير موافق	
					غير موافق بشدة	
					متى تعتقد انه يجب التوقف عن تناول المضاد الحيوي؟	Q16
					عندما تشعر بتحسن	
					عند الانتهاء من المضاد الحيوي كما هو موصوف من قبل الطبيب	
					لا اعلم	
		لا اعلم	لا	نعم	هل تعتقد ان الاعراض التالية يمكن علاجها بالمضادات الحيوية:	
					الحمى او ارتفاع درجة حرارة الجسم	Q17
					التهاب الحلق و اللوزتين	Q18
					الحصبة	Q19
					الم في الجسم	Q20
					الم بالرأس	Q21
					نقص المناعة المكتسب الايدز	Q22
					التهاب المسالك البولية	Q23
		لا اعلم	لا	نعم	هل سمعت بالمصطلحات التالية؟	
					مقاومة المضاد الحيوي	Q24

Q25	البكتيريا المقاومة للمضاد الحيوي					
Q26	البكتيريا الخارقة (Superbug bacteria)					
Q27	اين سمعت عن مصطلح مقاومة المضاد الحيوي؟					
	مزود خدمات طبية (طبيب، صيدلاني او ممرض ... الخ)					
	وسائل الاعلام (صحف او تلفاز او راديو او وسائل التواصل الاجتماعي)					
	صديق او قريب عائلي					
	لم اسمع عن مقاومة المضاد الحيوي من قبل					
		صحيح	خطأ			
Q28	مقاومة المضادات الحيوية هي مشكلة في دول اخرى و ليست مشكلة بالنسبة للأردن					
Q29	مقاومة المضادات الحيوية هي مشكلة من يتناول المضادات الحيوية بشكل متكرر فقط ولا علاقة للبقية بها					
Q30	البكتيريا المقاومة للمضادات الحيوية تستطيع الانتقال من شخص لاخر					
Q31	مشكلة مقاومة المضادات الحيوية يمكنها ان تؤثر علي و على افراد اسرتي					
Q32	تأثير الالتهابات الحاصلة من بكتيريا مقاومة للمضادات الحيوية على الاجراءات الطبية (مثل العمليات و نقل الاعضاء و علاج السرطان) اخطر و تهدد حياة المريض					
Q33	علاج البكتيريا المقاومة للمضادات الحيوية اكثر صعوبة					
Q34	البكتيريا المقاومة للمضادات الحيوية في تزايد مستمر					
Q35	تحصل المقاومة للمضادات الحيوية عندما يشكل جسم الانسان مقاومة للمضادات الحيوية و المضاد الحيوي لا يمكن ان يقوم بمفعوله بشكل جيد					
Q36	الاستخدام الغير مسؤول للمضادات الحيوية سبب رئيسي في ظهور البكتيريا المقاومة للمضادات الحيوية					
		موافق بشدة	موافق	محايد	غير موافق	غير موافق بشدة
Q37	يعتبر من الطبيعي تناول نفس المضاد الحيوي الذي تناوله صديق او قريب عائلي امتلك اعراض متشابهة					
Q38	على الاطباء وصف المضاد الحيوي فقط عند الحاجه له					
Q39	على الحكومة صرف مكافئة مالية لشركات الادوية التي تقوم بانتاج مضاد حيوي جديد					
Q40	على المزارعين اعطاء حيوانات المزرعة مضادات حيوية اقل					
Q41	يجب على الناس عدم الاحتفاظ بالمضادات الحيوية لغرض استخدامها في مرض لاحق					
Q42	يعتبر من الطبيعي شراء او طلب من الطبيب نفس المضاد حيوي في حال ان المضاد الحيوي ساعد على الشفاء في مرة سابقة					
Q43	يجب على الناس تناول المضادات الحيوية فقط عند وصفه من قبل طبيب					
Q44	على شركات الادوية تطوير مضادات حيوية جديدة					
Q45	تعتبر مقاومة المضادات الحيوية من اكبر المشاكل التي تواجه العالم					
Q46	سيحل الخبراء الطبيين مشكلة مقاومة المضادات					

	الحيوية قبل ما تصبح مشكلة خطيرة					
Q47	على الجميع ان يتحمل مسؤولية تناول المضادات الحيوية عند الحاجة					
Q48	انا لا يمكنني ان احدث اثر لوقف مقاومة المضادات الحيوية					
Q49	اشعر بالقلق جراء مقاومة المضادات الحيوية على صحتي و صحة عائلتي					
Q50	لا يوجد خطورة من المقاومة للمضادات الحيوية في حال تناولي المضاد الحيوي بشكل صحيح و حسب الوصفة الطبية					
		نعم	لا			
Q51	هل تعتقد انه يجب ان يكون هنالك حملات توعية مجتمعية لتثقيف المجتمع المحلي عن الاستخدام السليم للمضادات الحيوية؟					
Q52	هل انت مستعد للمشاركة وحضور حملات التوعية عن خطورة البكتيريا المقاومة للمضادات الحيوية؟					
Q53	هل تعلم ان من طرق منع انتشار البكتيريا المقاومة للمضادات الحيوية هي غسل اليدين و المطاعيم					
Q54	هل تعلم بوجود مصطلح الصحة الجمعية (One Health) وهو عبارة عن تداخل الانسان والحيوان والبيئة في الصحة العامة وتصب هذه الاطراف في التقليل من خطورة البكتيريا المقاومة للمضادات الحيوية					
Q55	هل قمت بتغيير نمط استخدامك للمضادات الحيوية بناءاً على وعيك بخطورة البكتيريا المقاومة للمضادات الحيوية					
Q56	ما هو سبب تناولك المضاد الحيوي بدون وصفة طبية؟					
	لمعالجة مرض					
	للوقاية من حصول مرض					
	تقليل تكاليف العلاج الطبي					
Q57	عادة، كيف تحصل على المعلومات عن المضاد الحيوي و كيفية استخدامه؟					
	جهات صحية مختصة					
	وسائل الاتصال (التلفاز والراديو والانترنت، الخ)					
	العائلة و الاصدقاء					
