# Supplementary material for: Antibiotic Knowledge and Non-Prescription Antibiotic Use Among Jordanian Adults: A Community-Based Cross-Sectional Study
Source: Antibiotics (Basel). 2026 Oct 14;15(10):1017. doi: 10.3390/antibiotics15101017 (PMC13700557; doi:10.3390/antibiotics15101017)
Supplement: Supplementary file 1 [file antibiotics-15-01017-s001.zip › TablesS1-S3.pdf]

Table S1: Regression table of the antibiotic knowledge score based on participants' knowledge of appropriate antibiotic indications.

Variable	Category	Crude OR (95% CI)	P value	Adjusted OR (95% CI)	P value
Antibiotic knowledge score	Per 1-point increase	1.023 (0.957–1.092)	0.505	1.006 (0.936–1.080)	0.881
Sex	Male	Reference			
	Female	0.982 (0.757–1.273)	0.888	0.985 (0.740–1.313)	0.921
Age (years)	18–30	Reference			
	31–40	1.171 (0.837–1.637)	0.357	1.116 (0.716–1.741)	0.627
	41–50	1.297 (0.933–1.801)	0.121	1.264 (0.787–2.030)	0.333
	51–60	1.827 (1.200–2.781)	**0.005**	1.872 (1.072–3.268)	**0.028**
	≥60	0.767 (0.379–1.555)	0.462	0.730 (0.331–1.610)	0.435
Region	Northern	Reference			
	Central	1.232 (0.861–1.762)	0.255	1.237 (0.846–1.808)	0.273
	Southern	0.669 (0.390–1.145)	0.143	0.632 (0.358–1.114)	0.113
Monthly income (JOD)	<300	Reference			
	300–600	1.210 (0.891–1.644)	0.221	1.151 (0.811–1.632)	0.432
	601–1,000	1.299 (0.903–1.869)	0.159	1.167 (0.754–1.808)	0.489
	1,000–2,000	1.139 (0.680–1.910)	0.621	1.031 (0.557–1.907)	0.923
	>2,000	1.197 (0.508–2.819)	0.681	1.138 (0.455–2.844)	0.783
Educational level	No education	Reference			
	High school	0.455 (0.230–0.900)	**0.024**	0.415 (0.196–0.879)	**0.022**
	Diploma	0.673 (0.342–1.323)	0.251	0.603 (0.285–1.277)	0.187
	Bachelor's degree	0.647 (0.347–1.207)	0.171	0.595 (0.290–1.219)	0.156
	Master's degree	0.461 (0.223–0.952)	**0.036**	0.411 (0.181–0.933)	**0.034**
	PhD	0.627 (0.268–1.469)	0.283	0.537 (0.200–1.442)	0.217
Marital status	Single	Reference			
	Married	1.216 (0.925–1.598)	0.162	0.914 (0.610–1.368)	0.661
	Divorced	1.108 (0.483–2.544)	0.808	0.776 (0.311–1.936)	0.587
	Widowed	1.478 (0.580–3.763)	0.413	1.200 (0.410–3.513)	0.74

Table S2: The model diagnostics table

Model assessment	Result
Total study sample	1,031
Complete-case regression sample	878
Hosmer–Lemeshow test	$\chi^2 = 5.52$, df = 8; P = 0.701
ROC AUC	0.591
McFadden pseudo-R^2	0.02
Overall likelihood-ratio test	P = 0.358
Primary knowledge score	aOR = 1.009 (95% CI 0.927–1.098); P = 0.840
Sensitivity analysis excluding sore throat/tonsillitis	aOR = 1.032 (95% CI 0.947–1.126); P = 0.473

Table S3: Model diagnostics/sensitivity table for sore throat evaluation

Variable	Category	Crude OR (95% CI)	P value	Adjusted OR (95% CI)	P value
6-item antibiotic knowledge score	Per 1-point increase	1.039 (0.971–1.111)	0.267	1.020 (0.948–1.098)	0.588
Sex	Male	Reference			
	Female	0.982 (0.757–1.273)	0.888	0.981 (0.736–1.307)	0.895
Age group (years)	18–30	Reference			
	31–40	1.171 (0.837–1.637)	0.357	1.115 (0.715–1.738)	0.632
	41–50	1.297 (0.933–1.801)	0.121	1.261 (0.785–2.025)	0.338
	51–60	1.827 (1.200–2.781)	**0.005**	1.877 (1.075–3.277)	**0.027**
	≥60	0.767 (0.379–1.555)	0.462	0.728 (0.330–1.606)	0.431
Region	Northern	Reference			
	Central	1.231 (0.861–1.762)	0.255	1.233 (0.843–1.802)	0.28
	Southern	0.669 (0.390–1.145)	0.143	0.631 (0.358–1.113)	0.112
Monthly income (JOD)	<300	Reference			
	300–600	1.210 (0.891–1.644)	0.221	1.138 (0.803–1.614)	0.468
	601–1,000	1.299 (0.902–1.869)	0.159	1.151 (0.743–1.784)	0.529
	1,000–2,000	1.139 (0.680–1.910)	0.621	1.019 (0.550–1.886)	0.953
	>2,000	1.197 (0.508–2.819)	0.681	1.120 (0.448–2.798)	0.808
Educational level	No formal education	Reference			
	High school	0.455 (0.230–0.900)	**0.024**	0.413 (0.195–0.874)	**0.021**
	Diploma	0.673 (0.342–1.323)	0.251	0.598 (0.282–1.267)	0.18
	Bachelor's degree	0.647 (0.347–1.207)	0.171	0.588 (0.287–1.205)	0.147
	Master's degree	0.461 (0.223–0.952)	**0.036**	0.407 (0.179–0.923)	**0.031**
	PhD	0.627 (0.267–1.469)	0.283	0.533 (0.198–1.432)	0.212
Marital status	Single	Reference			
	Married	1.216 (0.925–1.598)	0.162	0.913 (0.610–1.367)	0.658
	Divorced	1.108 (0.483–2.544)	0.808	0.774 (0.310–1.928)	0.582
	Widowed	1.478 (0.580–3.763)	0.413	1.190 (0.406–3.484)	0.751
